# ClpV3 of the H3-Type VI Secretion System (H3-T6SS) Affects Multiple Virulence Factors in *Pseudomonas aeruginosa*

**DOI:** 10.3389/fmicb.2020.01096

**Published:** 2020-05-29

**Authors:** Yanqi Li, Lin Chen, Pansong Zhang, Anjali Y. Bhagirath, Kangmin Duan

**Affiliations:** ^1^Department of Oral Biology, Rady Faculty of Health Sciences, University of Manitoba, Winnipeg, MB, Canada; ^2^College of Life Sciences, Northwest University, Xi’an, China; ^3^Department of Medical Microbiology & Infectious Diseases, Rady Faculty of Health Sciences, University of Manitoba, Winnipeg, MB, Canada

**Keywords:** *Pseudomonas aeruginosa*, secretion system, ClpV3, virulence factors, signal molecules

## Abstract

The type VI secretion system (T6SS) is a toxic effector delivery apparatus widely distributed in Gram-negative bacteria. The opportunistic pathogen *Pseudomonas aeruginosa* encodes three T6SSs, namely H1-, H2-, and H3-T6SS. Each T6SS possesses its own effectors and their roles are not yet fully understood. Here, we report that an H3-T6SS deletion mutant PAO1(Δ*clpV3*) significantly affected the virulence-related phenotypes including pyocyanin production, biofilm formation, proteolytic activity, and motilities. Most interestingly, the expression of T3SS genes was markedly affected, indicating a link between H3-T6SS and T3SS. RNA-Sequencing was performed to globally identify the genes differentially expressed when H3-T6SS was inactivated and the results obtained correlated well with the observed phenotypes. Interestingly, the expressions of T2SS, T3SS, H2-T6SS, and H3-T6SS were all significantly decreased, while H1-T6SS was increased in the PAO1(Δ*clpV3*) strain. We also observed that the intracellular concentration of secondary messenger cAMP was reduced in PAO1(Δ*clpV3*), and the c-di-GMP level was also decreased as indicated by the decreased *cdrA* reporter activity. Finally, by using a *Galleria mellonella* infection model, we show that H3-T6SS plays a key role in the pathogenicity of *P. aeruginosa in vivo*. Overall, our study highlights the unique connection of H3-T6SS in *P. aeruginosa* with T3SS, pyocyanin production, biofilm formation and *in vivo* pathogenicity.

## Introduction

*Pseudomonas aeruginosa* is an important human pathogen capable of growing in a wide range of environmental conditions (Arai, 2011). On the list of antibiotic-resistant “priority pathogens” published by World Health Organization, *P. aeruginosa* is classified as one of the critical pathogens that pose the greatest threat to human health (Willyard, 2017). *P. aeruginosa* especially affects patients who are immunocompromised or suffer with burn wounds, urinary tract infections, and cystic fibrosis (Mittal et al., 2009; Migiyama et al., 2016; Stefani et al., 2017). *P. aeruginosa* has an arsenal of virulence factors and pathogenic mechanisms, such as toxic protein secretion systems, quorum sensing systems (QS), biofilm formation, and antibiotic resistance (Lau et al., 2005; Bleves et al., 2010; Singh et al., 2017). Among these, the contact-dependent type VI secretion system (T6SS) directly translocates toxic effectors into prokaryotic or eukaryotic target cells (Ho et al., 2014; Jiang et al., 2014; Cianfanelli et al., 2016).

The structure of T6SS consists of a phage like tail as well as several sub-complexes that secrets toxins into target cells in a one-step manner (Bleves et al., 2010). Thirteen essential genes are conserved in all T6SSs (Filloux et al., 2008). These proteins named from TssA to TssM. TssJ, TssL, and TssM make up the membrane core complex that serves as a platform for baseplate assembly and a T6SS docking station (Durand et al., 2012; Logger et al., 2016). The T6SS baseplate complex is composed of TssE, TssF, TssG, and TssK, which facilitates the correct assembly of inner tubes and contractile sheath (Zoued et al., 2013; Brunet et al., 2015). Type VI secretion system sheath consists of two contractile proteins, TssB and TssC, forming tubular structures (Zoued et al., 2016; Gallique et al., 2017) while TssA forms a dodecamer complex, connecting the sheath structure to the membrane complex (Planamente et al., 2016). ClpV is a cytoplasmic AAA+ ATPase protein and is an essential component of T6SS (Schlieker et al., 2005). In *P. aeruginosa*, T6SS effector delivery is driven by ATP hydrolysis which generates the force for toxin secretion (Corbitt et al., 2018). Once the sheath is contracted, ClpV recognizes and interacts with TssC to disassemble the sheath, therefore, TssB and TssC can be reused for a new round of translocation. Planamente et al. (2016) demonstrated that ClpV interacts with TssA, suggesting ClpV is not only responsible for TssBC sheath disassembly, but is also involved in recycling other T6SS components.

There are three T6SSs in *P. aeruginosa*: H1-T6SS, H2-T6SS, and H3-T6SS. The expression of T6SSs in *P. aeruginosa* is regulated by the QS system (Lesic et al., 2009). There are several QS systems in *P. aeruginosa*, two N-acyl-homoserine lactone based QS systems (*las* and *rhl* systems) and one quinolone PQS system (*pqs*). The expression of H1-T6SS is negatively regulated by both *las* and *pqs* QS systems, while the expression of H2- and H3-T6SS is positively regulated by *las, rhl*, and *pqs* (Lesic et al., 2009; Sana et al., 2013). The RNA-binding post-transcriptional regulator RsmA represses all the T6SS clusters in *P. aeruginosa* (Allsopp et al., 2017). Extrinsic environmental factors play an important role in shaping pathogenesis and iron availability regulates a wealth of genes via ferric uptake regulator Fur (Pasqua et al., 2017). Iron has been shown to reduce the expression of H2- and H3-T6SSs (Sana et al., 2012, 2013; Lin et al., 2017).

The T6SSs in *P. aeruginosa* is not only involved in competition against other bacteria, but also survival, colonization and full virulence against the host (Sana et al., 2016). H1-T6SS is the most studied and only associated with killing prokaryotic cells, while H2- and H3-T6SS have been shown to target both bacterial and host cells but are poorly understood. H1-T6SS translocates at least seven effectors, Tse1-7, to kill prokaryotic preys (Pissaridou et al., 2018), whereas H2- and H3-T6SS target both bacterial and host cells, but only a few effectors have been identified. Recently, the role of the T6SSs has been shown to extend beyond the delivery of toxic effectors (Lin et al., 2017; Han et al., 2019). However, in depth studies on their roles are lacking. PldA, PldB, Tle3, Azu, and TplE are injected through H2-T6SS (Moore et al., 2013; Berni et al., 2019; Han et al., 2019; Wettstadt et al., 2019), while TseF is secreted by H3-T6SS (Lin et al., 2017). Azu, a Cu^2+^ binding protein, is secreted by H2-T6SS in *P. aeruginosa* for Cu^2+^ acquisition (Han et al., 2019). TseF dependent on H3-T6SS directly interacts with PQS and is incorporated with outer membrane vesicles (OMVs) for iron acquisition (Lin et al., 2017). Multiple T6SSs empower bacteria to better adapt and survive in the complicated polymicrobial communities not just limited to translocating toxic effectors into prey cells. Studies on T6SSs in other bacteria suggest a far more complex role. The T6SS-4 from *Yersinia pseudotuberculosis* is involved in the transportation of Zinc to permit the bacteria to survive oxidative stress as well as host immunity (Wang et al., 2015). Weber et al. (2009) have shown that the T6SS proteins from *Vibrio anguilarum* regulate expression of the stress response regulator RpoS and the quorum sensing regulator VanT, and suggest that T6SS could also function as a signal sensing system as well (Weber et al., 2009). A recent study demonstrated that the T6SS-4 in *Burkholderia thailandensis* plays a role in accumulating intracellular concentration of manganese through the Mn^2+^ binding effector (TseM) under oxidative stress (Si et al., 2017). Matthey et al. (2019) have shown that *V. cholerae* are able to acquire free DNA from their surroundings in a T6SS-dependent manner suggesting a role in anti-microbial resistance.

In this study, we constructed a *clpV3* deletion mutant in *P. aeruginosa* PAO1 and found that the virulence factors (pyocyanin production, biofilm formation, proteolytic activity, motility, and T3SS) were significantly affected by the impairment of H3-T6SS. Furthermore, we performed RNA-Sequencing to compare the globally transcriptional profiles between PAO1 and PAO1(Δ*clpV3*) and identified 311 differentially expressed genes (DEGs). The effect of H3-T6SS on *in vivo* pathogenicity was also examined by using a *Galleria mellonella* infection model.

## Materials and Methods

### Bacterial Strains, Plasmids, and Growth Conditions

The bacterial strains and plasmids used in this study are listed in Table 1. *P. aeruginosa* and *Escherichia coli* strains were grown in LB broth and agar plates at 37°C. The concentrations of antibiotic used were as follows: for *E. coli*, tetracycline (12.5 μg/ml), ampicillin (100 μg/ml), and kanamycin (50 μg/ml), and for *P. aeruginosa*, tetracycline (70 μg/ml), carbenicillin (250 μg/ml), and trimethoprim (300 μg/ml). For construction of *P. aeruginosa* mutant PAO1(Δ*clpV3*), tetracycline at 300 μg/ml in *Pseudomonas* isolation agar (PIA) was used for specific selection.

**TABLE 1:** Bacterial strains and plasmids used in this study.

**Bacterial strain or plasmid**	**Relevant characteristics/sequence**	**Source**
***E. coli* strains**		
DH5α	F^–^ φ80*lac*ZΔM15 Δ(*lacZYA*-*argF*) U169 *recA*1 *endA*1 *hsdR*17 (r_k_^–^, m_k_^+^) phoA supE44 λ^–^ *thi*^–^1 *gyrA*96 *relA*1	Invitrogen
SM10-λ *pir*	Mobilizing strain, RP4 integrated in the chromosome; Kn^r^	Simon et al., 1983
***P. aeruginosa* strains**		
PAO1	Wild type, lab strain	Holloway et al., 1994
PAO1(*ΔclpV3*)	*ClpV3* knockout mutant of PAO1	This study
**Plasmids**		
pMS402	Expression reporter plasmid carrying the promoterless *luxCDABE*; Km^r^ Tmp^r^	Duan et al., 2003
CTX-6.1	Integration plasmid origins of plasmid mini-CTX-*lux*; Tc^r^	Kong et al., 2013
pRK2013	Broad-host-range helper vector; Tra^+^, Km^r^	Ditta et al., 1980a
pEX18Tc	*oriT*^+^ *sacB*^+^ gene replacement vector with multiple-cloning site from pUC18; Tc^r^	Hoang et al., 1998
pAK1900	*E. coli*-*P. aeruginosa* shuttle cloning vector, Amp^r^	Sharp et al., 1996
pEX18Tc-*clpV3*_up_	pEX18Tc carrying the upstream fragment of *clpV3*	This study
pEX18Tc-*clpV3*_up+dw_	pEX18Tc carrying the upstream and downstream fragment of *clpV3*	This study
pAK-*clpV3*	pAK1900 with a 2774 bp fragment of *clpV3* between *Kpn*I and *Hin*dIII; Amp^r^, Cb^r^	This study
CTX-*phzA1*	Integration plasmid, CTX6.1 with a fragment of pKD-*phzA1* containing *phzA1* promoter region and *luxCDABE* gene; Km^r^, Tmp^r^, Tc^r^	Guo et al., 2016
CTX-*phzA2*	Integration plasmid, CTX6.1 with a fragment of pKD-*phzA2* containing *phzA2* promoter region and *luxCDABE* gene; Km^r^, Tmp^r^, Tc^r^	Guo et al., 2016
pKD -*exoY*	pMS402 containing *exoY* promoter region, Km^r^, Tmp^r^	Guo et al., 2016
pKD -*exsD*	pMS402 containing *exsD* promoter region, Km^r^, Tmp^r^	Kong et al., 2013
pKD-*cdrA*	pMS402 containing *cdrA* promoter region, Km^r^, Tmp^r^	Bhagirath et al., 2018

### Construction of *P. aeruginosa* PAO1(Δ*clpV3*) Mutant

To generate the *clpV3* deletion mutant PAO1(Δ*clpV3*), the pEX18Tc sucrose counter selection system was used for unmarked deletion of *clpV3* gene as described previously (Hoang et al., 1998; Hmelo et al., 2015). Briefly, the upstream and downstream fragment of *clpV3* were amplified by PCR with the primers *clpV3*-up-S/*clpV3*-up-AS and *clpV3*-dw-S/*clpV3*-dw-AS, respectively (Table 2). The upstream fragment firstly was cloned into the vector pEX18Tc treated with the same restriction enzyme yielding pEX18Tc-*clpV3*_up_. Following double restriction enzyme digestion (*Bam*HI and *Hin*dIII) as well as downstream fragment, these two products were ligated to generate pEX18Tc-*clpV3*_up+dw_. The PAO1(Δ*clpV3*) was obtained by means of tri-parental mating as described previously (Ditta et al., 1980b). In brief, overnight cultures of the donor strain *E. coli* containing the plasmid pEX18Tc-*clpV3*_up+dw_, the helper *E. coli* strain containing pRK2013 and the recipient PAO1 were collected and re-suspended in PBS. The bacteria were mixed in a ratio of 2:2:1 and then spotted onto LB agar plates. After culturing at 37°C overnight, the bacteria were scraped off and re-suspended in 500 μl of LB. The diluted suspensions were spread on PIA plates containing tetracycline at 300 μg/ml to select for merodiploids. After first crossover, the grown colony was streaked on no salt LB plate containing 10% sucrose to select for double crossover. The resultant *clpV3* knockout mutant was verified by PCR with the primers C-*clpV3*-S/C-*clpV3*-AS and designated as PAO1(Δ*clpV3*).

**TABLE 2:** Primers used in this study.

**Primer**	**Sequence (5′→3′)**	**Restriction site**
*clpV3*-up-S	TAGGAATTCGCCCTATGCCTACCAGGAA	*Eco*RI
*clpV3*-up-AS	TAAGGATCCGCAGGTGCTCGATCTCTACG	*Bam*HI
*clpV3*-dw-S	TACGGATCCTGGTGGTGGACTTCAGGAAC	*Bam*HI
*clpV3*-dw-AS	CCCAAGCTTTTGCTTTCTTCGCTTGTGAA	*Hin*dIII
C-*clpV3*-S	GGTGGAAAGCCTGCTCGACGAC	
C-*clpV3*-AS	GCGAGGATCCTTTGCCACTTGG	
pAK-*clpV3*-S	TATGGTACCGACCTGGATTGTCGCCTGA	*Kpn*I
pAK-*clpV3*-AS	CAGAAGCTTTCTTCGCTTGTGAATGGCAC	*Hin*dIII
*rpsL*-F	TCTGACCAACGGTTTCGAGG	
*rpsL*-R	GCCCGGAAGGTCCTTTACAC	
*rsmA*-F	GACGGTACTGGGTGTCAAAGGGAAC	
*rsmA*-R	CTCTTGATCTTTCTCTTTCTGGATGCG	
*exoS-*F	GCATCAGGTAATGAGCGAGGTCG	
*exoS-*R	GGCTGTCTGCCCAGGTACTTTTCC	
*phzA1-*F	CGGTCAGCGGTACAGGGAAACA	
*phzA1-*R	CGAACAGGCTGTGCCGCTGTA	
*phzA2-*F	GCGAGAGTACCAACGGTTGAAAGG	
*phzA2-*R	GAACAGGCTGTGCCGCTGTAAC	
*lasA-*F	CGCCATCCAACCTGATGCAAT	
*lasA-*R	CGTAGGACGCATCGAAGGACGA	
*lasR-*F	CTGTACCCAGAGCGTACTGCCGA	
*lasR-*R	CGGCATGGTCAGCCCATACAC	

*Underlined are restriction site sequences.*

### Measurement of Pyocyanin Production

Supernatants from 18 h incubation bacterial culture were collected to extract and quantify the pyocyanin production according to the previously described methods (Essar et al., 1990). Briefly, 5 ml of the culture supernatant was fully mixed with 3 ml of chloroform, then the chloroform layer was transferred to a new tube containing 1 ml of 0.2 N HCl. After centrifugation at 4500 *g* for 10 min, the top layer was transferred to cuvette to measure its absorbance at 520 nm. The concentrations obtained, expressed as micrograms of pyocyanin produced per milliliter of culture supernatant, were calculated by multiplying the extinction coefficient of 17.072 at 520 nm.

### Measurement of Promoter Activities

The procedures of the *lux*-based reporter assays were described previously (Duan et al., 2003; Bhagirath et al., 2017). In brief, bacteria were incubated overnight in LB broth followed by sub-inoculating into fresh medium to OD_600_ of 0.2 and cultivated for an additional 3 h before use as inoculants. The cultures were inoculated into 96-well plates with transparent bottom in triplicates in a ratio of 5 μl of inoculum to 95 μl of fresh medium. 50 μl of filter-sterilized mineral oil (Sigma Aldrich) was added on top to prevent evaporation during the assay. Luminescence (counts per second, cps) was measured every 30 min for 24 h in a Synergy H4 Multimode Microplate Reader (BioTek). Bacterial growth was monitored at the same time by measuring OD_600_. The level of gene expression was normalized to bacterial growth and is presented as cps/OD_600_.

### Assays for Biofilm Formation

Biofilm production was quantified as previously described (O’Toole, 2011). Cells from overnight cultures were diluted at 1:100 into M63 minimal medium supplemented with magnesium sulfate, glucose and casamino acids, then inoculated in 96-well polystyrene microtiter plates (Costar) and grown at 37°C for 24 h. After incubation, the cells were discarded, and the plate was gently submerged in a small tub of water to remove unattached cells and media components. A 125 μl volume of 0.1% crystal violet was added to each well and staining was allowed for 20 min at room temperature. Wells were rinsed three times with distilled water, and 125 μl of 30% acetic acid in water was added to dissolve the remaining crystal violet. A 100 μl portion of this solution was transferred to a new plate, and the absorbance was measured at 550 nm (OD_550_).

### Measurement of Proteolytic Activity

Skim milk proteolysis was determined through the use of agar plate assays as described in the previous study with minor modification (Gupta et al., 2009). One microliter of cells from overnight culture were inoculated on LB plate containing 2% skim milk and grown at 37°C for overnight. Zones of clearance surrounding the bacterial colonies indicate proteolytic activity and the sizes of the zones were measured.

### Swarming and Swimming Motility Examination

Bacterial motility activities were assessed as described previously (Rashid and Kornberg, 2000). Medium used for swarming assay consisted of 8 g/l nutrient broth, 5 g/l glucose and 0.5% (wt/vol) agar. For swimming assay, the medium contained 10 g/l tryptone, 5 g/l NaCl and 0.3% agar. For swarming and swimming motilities, bacteria were spotted onto plates as a 1 μl of aliquot taken directly from overnight LB cultures. After inoculation, photographs were acquired with the Fusion FX7 Vilber Lourmat Imaging machine.

### Quantification of Intracellular cAMP Levels

Intracellular concentration of cAMP was quantified by using Cyclic AMP Select ELISA kit (Cayman Chemical, United States). Overnight culture of 8 OD_600_ units were pelleted by centrifugation and mixed with 500 μl of 0.1 N HCl. Following 30 s of sonication, the supernatants were collected by centrifugation at 1000 g for 10 min and transferred to a fresh tube. According to the manufacturer’s protocol, the standards and samples were prepared and loaded into the 96-well supplied plate. After incubation and development of the plate, wavelengths between 405 and 420 nm were read. The concentration of each sample was determined by using the equation obtained from the standard curve plot.

### Complementation of the *clpV3* Knockout Mutant

For the complementation experiments, The *E. coli*-*P. aeruginosa* shuttle vector pAK1900 was used (Sharp et al., 1996). *clpV3* gene was generated by PCR with the primers pAK-*clpV3*-S/pAK-*clpV3*-AS listed in Table 2. This PCR amplified fragment was cloned into PAK1900 and the resultant plasmid pAK-*clpV3* was transformed into PAO1(Δ*clpV3*) by electroporation.

### RNA Isolation

Strains were grown overnight at 37°C followed by sub-culturing into fresh medium and grown to mid-exponential phase. Total RNA was extracted by TRIzol-based method (Life Technologies, CA, United States). In brief, the cultures were centrifuged at 12,000 *g* for 5 min. The supernatant was discarded, cell pellets were resuspended in 1 ml of TRIzol and then incubated at room temperature for 5 min to permit complete dissociation of nucleoproteins complex. Followed by adding 0.2 ml of chloroform into the tube and mix well, the samples were centrifuged (12,000 *g* at 4°C for 15 min) to form three layers. The upper aqueous phase was transferred to a fresh tube and added by 0.5 ml of cold isopropanol to precipitate RNA. After centrifugation, the supernatant was removed, and the pellet was suspended in 1 ml of 75% ethanol. Samples were centrifuged and the supernatant was discarded, air-dried RNA pellet was resolved in 50 μl of RNase-free Water.

### RNA-Seq Library Construction and Sequencing

RNA integrity was measured using Bioanalyzer 2100 (Agilent, Santa Clara, CA, United States) and rRNA was removed from 1 mg of total RNA with Ribo-Zero Magnetic Gold Kit (Epicentre Biotechnologies, Madison, WI, United States). To construct the RNA-Seq library, TruSeq RNA Sample Prep Kit v2 (Illumina, San Diego, CA, United States) was used. rRNA-depleted RNA was fragmented into small pieces using Elute Prime Fragment Mix. First-strand cDNA was synthesized with First Strand Master Mix and Super Script II reverse transcriptase (Invitrogen, Carlsbad, CA, United States). Following purification by Agencourt RNAClean XP beads (Beckman Coulter, CA, United States), the second-strand cDNA library was synthesized using Second Strand Master Mix and dATP, dGTP, dCTP, dUTP mix. Purified fragmented cDNA was end repaired (30 min at 37°C) prior to ligating sequencing adapters. Amplified RNA-Seq libraries were purified by using AMPureXP Beads. The clustering of the index-coded samples was performed on a cBot Cluster Generation System following to the manufacturer’s instructions, and the sequencing was performed using the Illumina Hiseq TM 2500 platform with pair-end 150 base reads.

### Bioinformatics Analysis

After RNA-Sequencing, the obtained raw data were filtered according to the following standards: (1) removing reads with ≥10% unidentified nucleotides (N); (2) removing reads with >50% bases having Phred quality scores of ≤20; (3) removing reads aligned to the barcode adapter using FASTP^1^. Quality trimmed reads were aligned using Bowtie2 (Langmead and Salzberg, 2012) (version 2.2.8) to the *P. aeruginosa* PAO1 reference genome to identify known genes and calculated gene expression by RSEM (Li and Dewey, 2011). The gene expression level was calculated and further normalized by using the fragments per kb of transcript per million (FPKM) mapped reads method to eliminate the influence of different gene lengths and amount of sequencing data on the calculation of gene expression. The edgeR package^2^ was used to identify DEGs across samples with fold changes ≥2 and a false discovery rate-adjusted P (*q* value) <0.05. Go terms and KEGG pathway were defined as being significantly enriched when the *q* value ≤0.05.

### Synthesis of cDNA and Quantitative Real-Time PCR

cDNA was generated from 1 μg total RNA using a Quanta qScript cDNA Synthesis kit (Quanta BioSciences, MD, United States). *rpsL* was used as housekeeping gene control and the primers were listed in Table 2. The qPCR was performed using PowerUp^TM^ SYBR^TM^ Green Master Mix (Thermo Fisher Scientific) on an Eco Illumina real-time detection system (Montreal Biotech) under the following conditions: UDG activation at 50°C for 2 min, Dual-Lock^TM^ DNA polymerase initiation at 95°C for 2 min, and 40 cycles of 95°C for 15 s and 60°C for 1 min. Fold changes of gene expression levels were calculated according to the 2^–ΔΔCq^ method. Each sample was measured in triplicate and repeated at least three times.

### *Galleria mellonella* Killing Assays

The *G. mellonella* infection model is a widely-accepted animal model and the experiments were performed as previously described (Desbois and Coote, 2011). The larvae were stored in wood chips at 10°C and used within two weeks from shipment. Prior to inoculation into *G. mellonella* caterpillars, *P. aeruginosa* cells were washed twice with PBS and then diluted in PBS to a final concentration of 1000 CFU/ml. A 10 μl Hamilton syringe was used to inject 10 μl of bacterial suspension into *G. mellonella* via the last left proleg. The infected larvae were incubated in a static incubator in the dark at 30°C, the optimum temperature for insect growth and development (Brennan et al., 2002). The number of dead caterpillars was scored at each time point. Caterpillars were considered dead when they displayed no movement in response to touch.

## Results

### Inactivation of H3-T6SS Altered Multiple *P. aeruginosa* PAO1 Phenotypes

**FIGURE 1:**
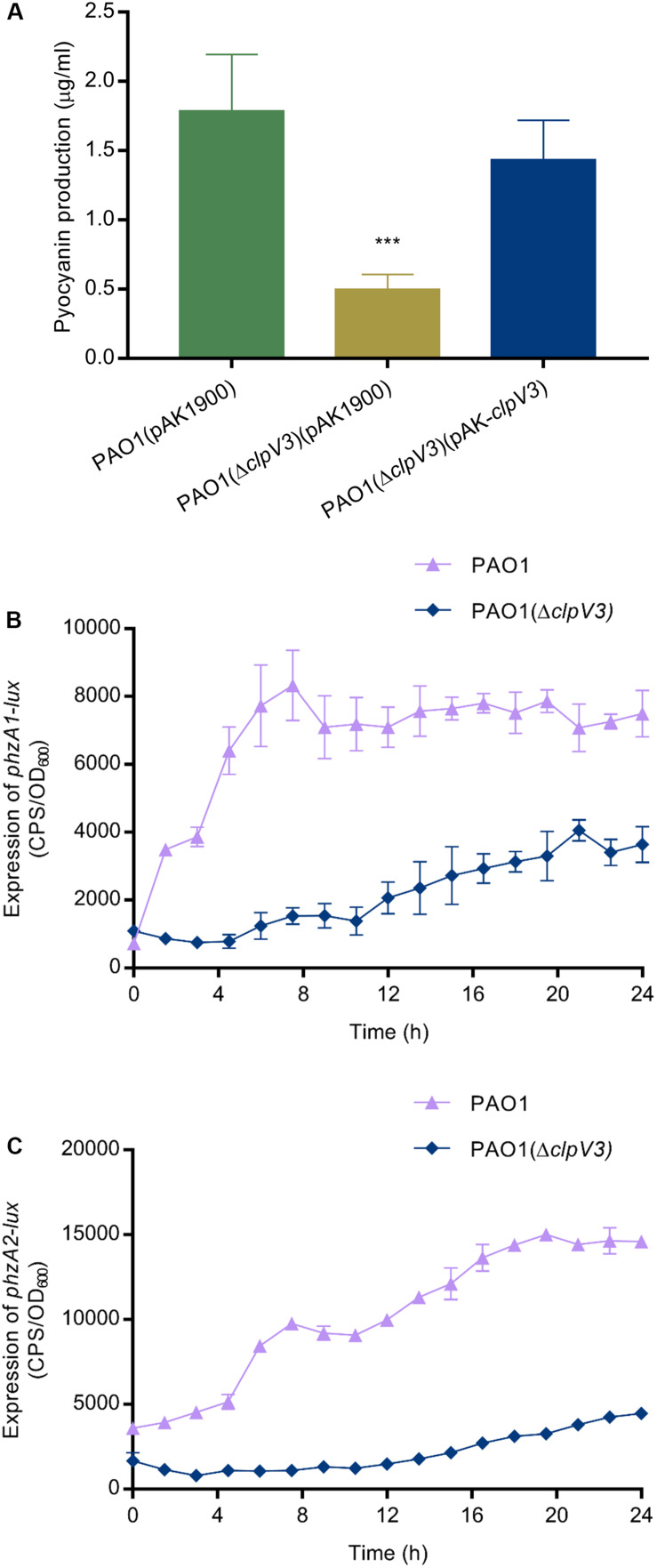
Reduced pyocyanin production and decreased expression of *phzA1* and *phzA2* in PAO1(Δ*clpV3*). **(A)** Comparison of pyocyanin production in PAO1(Δ*clpV3*),the wild-type PAO1 and the complementation strain. The experiment was independently performed three times. Unpaired Student’s *t*-test was used to analyze the data. ***p < 0.001. **(B,C)** Reduced promoter activities of two pyocyanin synthesis gene clusters *phzA1* and *phzA2* in PAO1(Δ*clpV3*). The promoter activity is presented as light production (counts per second, cps) normalized to OD_600_. The experiment was independently performed three times. Error bars indicate standard deviations.

In light of the non-competition functions of T6SSs and the fact that H3-T6SS is poorly understood, we constructed an H3-T6SS mutant PAO1(Δ*clpV3*) and examined virulence-related phenotypical changes in the mutant. Deletion of *clpV3* completely inactivates the function of H3-T6SS (Sana et al., 2013). We compared the virulence-related phenotypes between the wild type and the *clpV3* knockout mutant. A striking difference that was easily observed was the color change of the bacterial cultures. Measurement of pyocyanin production indicated that when compared with the wild-type PAO1 pyocyanin was significantly reduced in PAO1(Δ*clpV3*). Overexpression of *clpV3* gene in the PAO1(Δ*clpV3*) strain restored the pyocyanin production to the wild-type level (Figure 1A). Since two homologous operons are associated with synthesizing phenazine compounds in *P. aeruginosa*, *phzA1B1C1D1G1* (*phzA1*) and *phzA2B2C2D2G2* (*phzA2*) (Mavrodi et al., 2001), the reduced pyocyanin production in PAO1(Δ*clpV3*) strain led us to analyze the promoter activities of these two operons. As shown in Figures 1B,C, the *phzA1* and *phzA2* promoter activities in the PAO1(Δ*clpV3*) were 3-fold lower than in the wild-type PAO1, indicating the reduced pyocyanin production in the mutant was probably a result of the reduced transcription of these operons.

**FIGURE 2:**
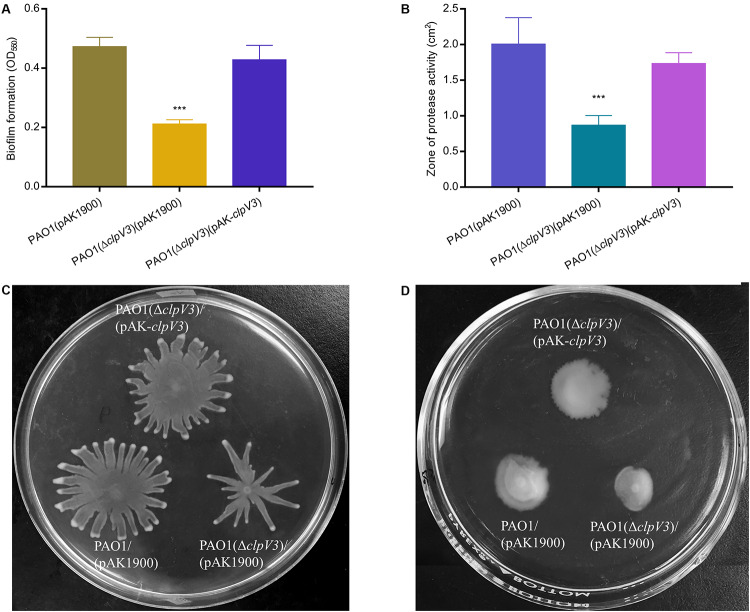
Inactivation of H3-T6SS in *P. aeruginosa* alters numerous phenotypes. **(A)** Biofilm formation was evaluated in 96-well microtiter plates and our results showed that biofilm formation was significantly decreased in PAO1(Δ*clpV3*) compared with in PAO1 or the complementation strain. **(B)** PAO1(Δ*clpV3*) showed smaller proteolytic zone than PAO1, which indicated that deletion of *clpV3* leads to producing less protease production. **(C,D)** Inactivation of H3-T6SS resulted in altered swarming motility and reduced swimming motility. The experiment was independently performed three times. Unpaired Student’s *t*-test was used to analyze the data. Error bars indicate standard deviations. ***p < 0.001.

We further examined the effect of H3-T6SS on other virulence factors including proteolytic activity, biofilm formation and bacterial motility. Proteases in *P. aeruginosa* play two important roles: subverting host immune responses and mediating host-directed damage (Laarman et al., 2012). Biofilms function as a protective barrier and allow bacteria to survive from the antimicrobial agents and the host immune systems (Drenkard, 2003; Haussler and Parsek, 2010). Motility is a key feature of acute phase of infection and is regulated by RsmA among other regulators (Heurlier et al., 2004). As shown in Figure 2, the results obtained demonstrated that biofilm formation was significantly decreased in PAO1(Δ*clpV3*) as compared to the wild-type PAO1 or the complemented strain (Figure 2A) and so was protease production (Figure 2B). In addition, PAO1(Δ*clpV3*) exhibited altered swarming patterns characterized by less and short tendrils compared with the pattern of wild type or the complemented strain (Figure 2C and Supplementary Figure S1A). Inactivation of H3-T6SS resulted in reduced swimming motility as well (Figure 2D and Supplementary Figure S1B). These results clearly indicate that the functionality of H3-T6SS is intricately connected with key virulence factors in *P. aeruginosa*.

### Deletion of *clpV3* Resulted in Decreased Expression of *exoY* and *exsD*

**FIGURE 3:**
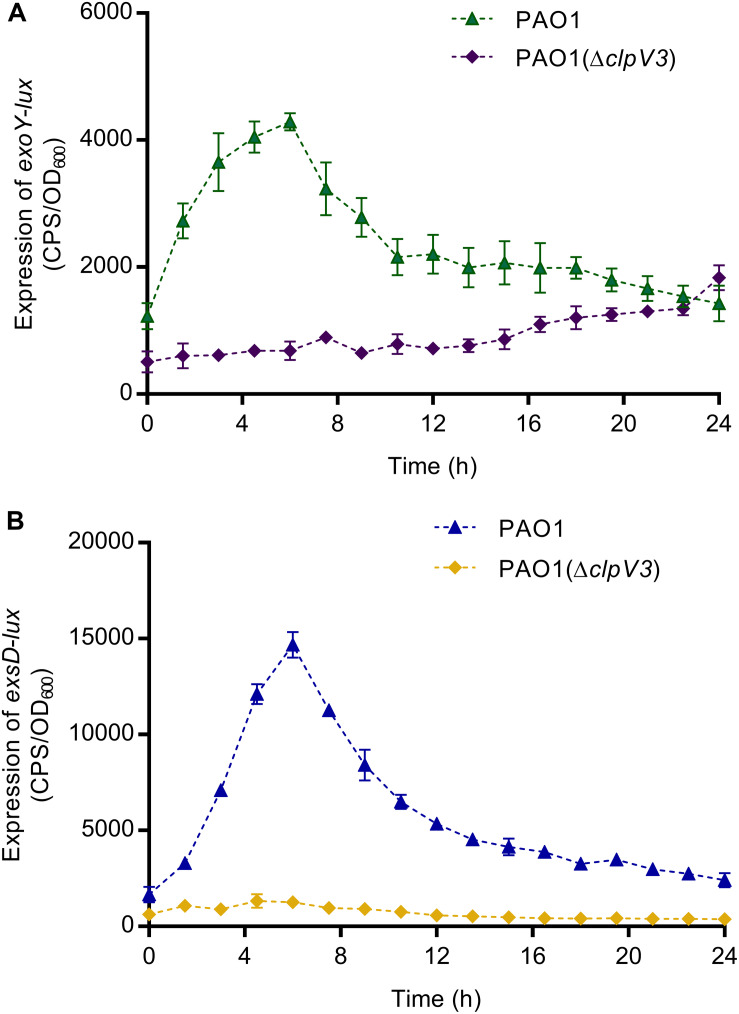
The promoter activities of *exoY* **(A)** and *exsD* **(B)** were significantly decreased in PAO1(Δ*clpV3*) when compared with in wild-type PAO1. The results clearly indicate that the expression of T3SS was significantly reduced when H3-T6SS is inactivated.

*Pseudomonas aeruginosa* utilizes a complex type III secretion apparatus to inject effector proteins (ExoS, ExoY, ExoT, and ExoU) into the host cells (Yan et al., 2019). T3SS and T6SS have been shown to be oppositely regulated (Moscoso et al., 2011) and both can target host cells. To find out whether the expression of T3SS was also affected in PAO1(Δ*clpV3*), the promoter activities of T3SS genes, including effector (*exoY*) and the *exsD-pscBCDEFGHIJKL* operon were analyzed in PAO1 and PAO1(Δ*clpV3*) using the *lux*-based pKD-*exoY* and pKD-*exsD* reporters. As shown in Figures 3A,B, the promoter activities of *exoY* and *exsD* were significantly decreased in PAO1(Δ*clpV3*) when compared with those in the wild-type PAO1, indicating that inactivation of H3-T6SS caused a significant reduction of the expression of T3SS genes.

### PAO1(Δ*clpV3*) Had Reduced Levels of Second Messengers c-di-GMP and cAMP

**FIGURE 4:**
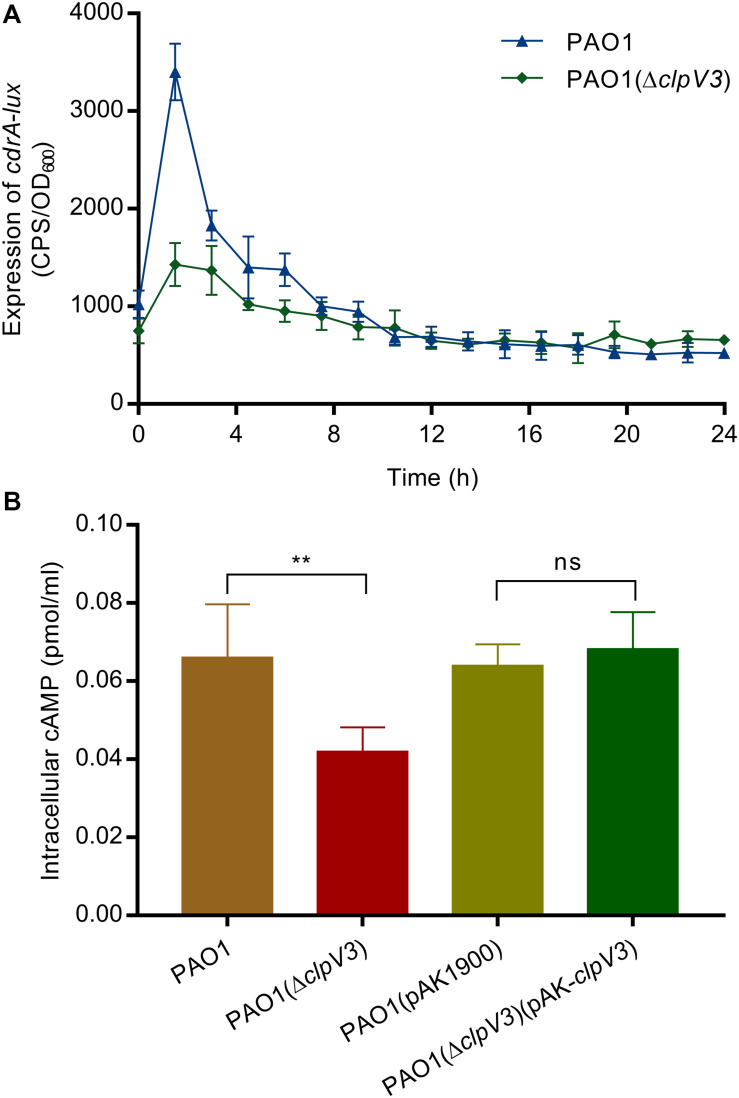
Intracellular concentration of secondary messengers (c-di-GMP and cAMP) were significantly reduced when *clpV3* gene was deleted. **(A)** PAO1(Δ*clpV3*) exhibited decreased *cdrA* promoter activity when compared with that of the wild-type PAO1, indicating a clear effect of H3-T6SS on intracellular c-di-GMP levels. **(B)** cAMP concentration was quantified in wild-type PAO1, PAO1(Δ*clpV3*) mutant and its complimented strain by using the Cyclic AMP Select ELISA Kit. Lower expressed cAMP was detected in *clpV3* deletion mutant compared with either the wild type or its complemented strain. Unpaired Student’s *t*-test was used to analyze the data, and the means and standard deviations are shown. **p < 0.01, ns, not significant.

Signaling molecules cyclic-di-GMP (c-di-GMP) and cyclic AMP (cAMP) play an important role in virulence factor regulation in *P. aeruginosa* (Almblad et al., 2015). c-di-GMP is a second messenger used by *P. aeruginosa* and other bacteria to regulate the expression of genes associated with flagella motility, Type IV pili and biofilm initiation (Valentini and Filloux, 2019). To investigate if the observed phenotypical changes could be a result of altered c-di-GMP levels, we tested *cdrA* promoter activity in the *clpV3* knockout mutant. The *cdrA* promoter activity levels have been shown to faithfully reflect the fluctuations in intracellular c-di-GMP levels (Rybtke et al., 2012). Quantifications of *cdrA* promotor activity in PAO1(Δ*clpV3*) and wild-type PAO1 were performed in triplicates and the data obtained are presented in Figure 4A. Compared with the wild-type PAO1, a decreased *cdrA* promoter activity was observed in PAO1(Δ*clpV3*), indicating a potential negative effect of H3-T6SS impairment on intracellular c-di-GMP levels. To determine if *clpV3* deletion affected cAMP levels in *P. aeruginosa*, the concentration of intracellular cAMP was measured for PAO1 and PAO1(Δ*clpV3*) using the Cyclic AMP Select ELISA Kit (Cayman Chemical Company, USA). As shown in Figure 4B, the *clpV3* mutant had significantly lower cellular cAMP than the wild-type PAO1, suggesting that the *clpV3* or the functionality of H3-T6SS contributes to the balance of *in vivo* cAMP concentrations. The level of cAMP in PAO1(Δ*clpV3*) was restored to the wild-type level upon complementation of *clpV3* on a plasmid. The decreased intracellular concentrations of the second messengers c-di-GMP and cAMP in the *clpV3* deletion mutant may explain, at least partially, the altered virulence factor production in this H3-T6SS mutant observed.

### Transcriptional Profiling of the *clpV3* Deletion Mutant

**FIGURE 5:**
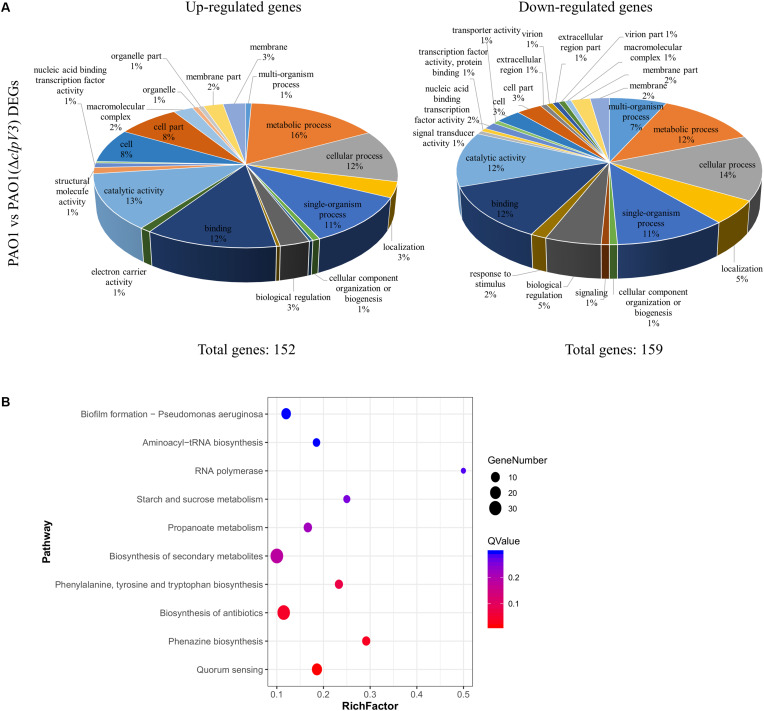
Comparison of the global transcriptomes of PAO1 and PAO1(Δ*clpv3*) by RNA-Seq. **(A)** Totally 311 genes were significantly differentially expressed in PAO1(Δ*clpV3*) compared to wild-type PAO1 with absolute fold change >2 and FDR <0.05. Pie charts showing Gene Ontology analysis of the differentially expressed genes outlined in the PAO1 vs PAO1(Δ*clpV3*), subdivided into up-regulated (left) and down-regulated (right) groups. **(B)** The top 10 of significant KEGG enrichments between PAO1 and PAO1(Δ*clpV3*).

It was apparent that the H3-T6SS mutant produced a reduced amount of virulence factors, such as pyocyanin. In order to determine globally the effect of *clpV3* inactivation, we performed RNA sequencing. The mRNAs were extracted from the culture grown in LB medium at the mid-exponential phase and the transcriptional profiles of PAO1(Δ*clpV3*) and wild-type PAO1 were compared. We found 311 significantly affected genes that were differentially expressed in these two strains. Hundred and fifty-two genes were up-regulated in *clpV3* deletion mutant and 159 down-regulated. The DEGs were assigned to 29 functional groups based on sequence homology. The significantly enriched (*q* < *0.05*) gene ontology (GO) categories among these DEGs are shown in Figure 5A and Supplementary Table S1. Comparing the expression patterns between PAO1 and PAO1(Δ*clpV3*), we were able to identify DEGs participating in quorum sensing, phenazine biosynthesis, biosynthesis of antibiotics, phenylalanine, tyrosine, and tryptophan biosynthesis, biosynthesis of secondary metabolism, and biofilm formation (Figure 5B).

It is observable that in PAO1(Δ*clpV3*) the expression of genes related with T2SS, T3SS, and pyocyanin synthesis were significantly down regulated. The specific virulence factors and regulatory pathways are grouped and listed in Supplementary Table S2. Such results are in agreement with the observation of reduced pyocyanin production, proteolytic activity, and lower promoter activities of T3SS in the mutant. Interestingly, all three T6SSs in *P. aeruginosa* were differently expressed when *clpV3* was deleted. The expression of both H2- and H3-T6SS was significantly decreased, compared with the wild-type PAO1, while the expression of H1-T6SS was significantly increased in PAO1(Δ*clpV3*). In addition, the RNA level of the post-transcriptional regulator RsmA which positively regulates T3SS and negatively regulates T6SSs was also lower in PAO1(Δ*clpV3*). Although RsmA activity is regulated mostly by small RNAs, such as RsmY and RsmZ, the altered transcription level of *rsmA* could also result in changed functionality of this regulator, The reduced expression of T3SS and increased H1-T6SS could, therefore, be due to the lower *rsmA* expression, at least partially. A previous study has shown that QS positively regulates H2- and H3-T6SS, but negatively regulates H1-T6SS (Lesic et al., 2009). We noted that the expression of several transcriptional regulators including LasR, VqsR, MexL, MvaT, and PauR were significantly decreased, while PmpR, CgrC, CgrB, PtxS, AntR, NarL, and Zur were significantly increased in the H3-T6SS mutant (Supplementary Table S2). The regulators that showed changed expression in the PAO1(Δ*clpV3*) potentially resulted in the lower expression of H2- and H3-T6SS, T2SS and T3SS and higher expression of H1-T6SS, which were also shown by the RNA-Sequencing analysis. Taken together, these results demonstrated that H3-T6SS impairment not only affected other T6SSs but also several genes involved in virulence and metabolic regulation in *P. aeruginosa*.

### Confirmation of the RNA-Sequencing Results With Selected Genes

**FIGURE 6:**
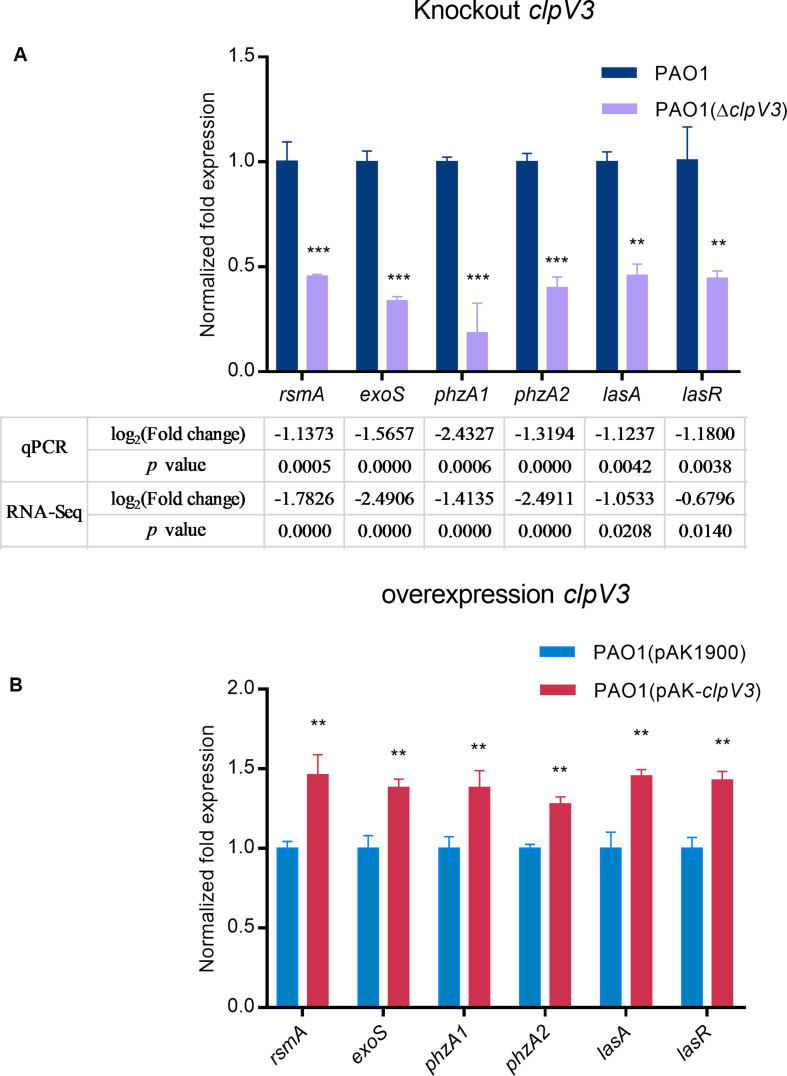
qPCR results after knocking down *clpV3* **(A)** and after overexpression of *clpV3* in PAO1 **(B)**. Assays were performed in triplicate. Statistical analysis was performed by unpaired Student’s *t*-test. ***p < 0.001; **p < 0.01.

RT-qPCR was carried out with selected genes to verify the RNA-Sequencing data. cDNA was prepared using the same RNA that was used for RNA-Sequencing. Genes selected for validation included *rsmA*, *exoS*, *phzA1*, *phzA2*, *lasA*, and *lasR*. Gene expression relative to housekeeping gene (*rpsL*) levels was calculated and shown in Figure 6A. The results showed decreased expression of these genes, in agreement with the RNA-Seq results. Furthermore, we constructed a *clpV3* overexpression strain and measured the expression of these previous tested genes. As shown in Figure 6B, the expression of these genes was significantly increased when *clpV3* was overexpressed. *P. aeruginosa* PAO1 strain carrying an empty vector PAO1(pAK1900) was used as a control in the comparison.

### The Effect of *clpV3* Deletion on *P. aeruginosa* PAO1 Virulence *in vivo*

**FIGURE 7:**
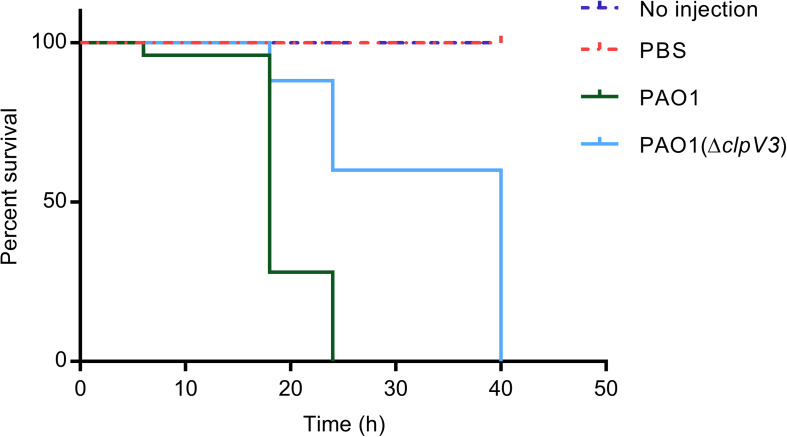
The effect of H3-T6SS on the pathogenicity of *P. aeruginosa in vivo*. The relative survival rates of the infected *G. mellonella* larvae were compared. PAO1(Δ*clpV3*) exhibited significantly increased survival rate compared with the wild-type PAO1. The experiment was performed three times on different days.

To verify the effect of the H3-T6SS on *P. aeruginosa* PAO1 pathogenicity *in vivo*, we used a *G. mellonella* infection model to examine the pathogenicity of PAO1(Δ*clpV3*). The relative survival rates of the infected *G. mellonella* larvae (*n* = 25) were compared between the *clpV3* mutant and the wild-type PAO1. As shown in Figure 7, the PAO1(Δ*clpV3*) infected larvae exhibited significantly increased survival rate compared with those infected with the wild-type PAO1. The decreased pathogenicity can be attributed to the decreased expression of virulence factors, such as pyocyanin production, T3SS, H2- and H3-T6SS. Our results, taken collectively, shown that H3-T6SS plays an important role in *P. aeruginosa* pathogenicity *in vivo* and may serve as a potential therapeutic target against its infection.

## Discussion

T6SS is a macromolecular weapon that is present in more than 25% of Gram-negative bacteria (Bingle et al., 2008). Multiple distinct T6SSs have been identified in a given bacterium including *P. aeruginosa*, suggesting T6SS’ versatile roles or specificities for particular niches. Distinct T6SSs might be activated in response to different environmental cues and hence have the extended functions beyond the delivery of toxic effectors (Bernard et al., 2010).

The results we obtained showed that deletion of *clpV3* in *P. aeruginosa*, which abolishes the functionality of H3-T6SS (Sana et al., 2013), dramatically affected phenotypes associated with pathogenicity. This is the first report that H3-T6SS affects virulence factors and biofilm formation in *P. aeruginosa*, which supports a role of H3-T6SS beyond the function of a weapon in competition against other microorganisms and/or killing host cells. In the *clpV3* deletion mutant, multiple virulence-related phenotypes were altered which included pyocyanin production, proteolytic activity, motilities, T3SS, and biofilm formation. Interestingly all of them were significantly reduced when H3-T6SS was inactivated by deletion of *clpV3*.

Among the virulence factors affected, pyocyanin is used by *P. aeruginosa* for interspecies competition and combating the host immune systems. It kills other competitors residing in the same niche and is able to elicit host responses by inactivating catalases known to protect against reactive oxygen species (ROS; Wilson et al., 1988; Ozyurek et al., 2011). Motility (swarming and swimming) is a key feature of *P. aeruginosa* during the acute infection phase and is also strongly associated with *P. aeruginosa* pathogenesis, as it enables colonization of different environments, attachment to surfaces, and formation of biofilm (Arora et al., 2005). Swarming is characterized as coordinated group activity and the motility pattern is dependent of the surfactant rhamnolipids (Caiazza et al., 2005; Tremblay et al., 2007). RhlAB are involved in the synthesis of rhamnolipids (Deziel et al., 2003), and the expression of *rhlA* and *rhlB* was reduced by 4.51- and 3.20-fold, respectively observed in PAO1(Δ*clpV3*) compared with that in wild-type PAO1 by RNA-Sequencing analysis (Supplementary Table S1). The changed motility in *clpV3* mutant is in agreement with the recent reported study that deletion of *clpV1* decreased swarming motility (Chen et al., 2020). Alkaline protease in *P. aeruginosa* is not only important for its virulence but is also important for immune evasion, though the exact mechanism is yet unknown (Laarman et al., 2012). We observed a decrease in proteolytic activity in *clpV3* mutant compared to the wild-type PAO1 strain. Thus, the inactivation of H3-T6SS not only abolished the function of H3-T6SS as a weapon against competitors and/or the host but also impaired other traits that are involved in such actions against competitors or the hosts.

Another important consequence of inactivation of H3-T6SS is the decreased biofilm formation. Biofilms function as both a structural scaffold and protective barrier to prevent access of antimicrobial agents and host immune identification (Drenkard, 2003; Haussler and Parsek, 2010). A recent study done by Chen et al. (2020) found that the *H1-T6SS*, *H3-T6SS* and *lasR* were expressed at higher levels in strong biofilm forming clinical isolates than in non-biofilm forming groups. Our observation that the *clpV3* mutant formed less biofilms than the wild type is in agreement with the reported findings suggesting a clinical relevance of the affected biofilm formation by H3-T6SS in *P. aeruginosa*.

T3SS is a system mainly used for interacting with eukaryotic host cells. T3SS and T6SS systems are reversibly regulated by the RsmA regulatory pathway (Brencic and Lory, 2009; Moscoso et al., 2011). The observation in our study that the expression of T3SS related genes (*exoY* and *exsD-pscBCDEFGHIJKL*) was significantly decreased in the *clpV3* mutant is the first report of a direct effect of T6SS on T3SS expression. Although common regulators have been reported such as RsmA that regulate both T6SS and T3SS, to our knowledge, no effect of T6SS has been reported to cause a change in T3SS.

Secondary messengers (cAMP and c-di-GMP) play important roles in regulating multiple phenotypical characteristics, and c-di-GMP has been shown to regulate multiple phenotypes including motility and biofilm formation (Moscoso et al., 2011). We measured these two secondary messengers and found a decreased level of cAMP in the *clpV3* mutant. This decreased cAMP level in *clpV3* mutant could explain the lower expression of T3SS because the genes involved in this secretion system are regulated by Vfr coupled with cAMP (Marsden et al., 2016). The other secondary messenger molecule c-di-GMP is capable of regulating the life styles of bacteria and controlling many key virulence factors (Valentini and Filloux, 2019). As compared to PAO1, the *clpV3* deletion mutant demonstrated a decrease in *cdrA* promoter activity, suggesting a reduced c-di-GMP level in the mutant. The decreased c-di-GMP signal molecules could hypothetically provide an explanation about the reduced virulence factors including pyocyanin production, proteolytic activity and biofilm formation (Hengge, 2009; Lo et al., 2016; Chang, 2017).

In an effort to globally investigate the effect of ClpV3, RNA-sequencing was performed to compare the DEGs between PAO1 and PAO1(Δ*clpV3*). The transcriptomic data revealed that a total of 311 genes were differentially expressed between the two strains. In a *clpV3* mutant, lower expression of genes related to pyocyanin synthesis, T2SS, T3SS, H2-T6SS, H3-T6SS was observed while an increased expression of H1-T6SS was also noted. These results correlate well with the observed phenotypic changes in the mutant. In addition, a number of transcriptional or post-transcriptional regulators were differentially expression, including *rsmA, vqsR*, *mexL*, *mvaT*, *pauR*, *pmpR*, *cgrC*, *cgrB*, *ptxS*, *antR*, *narL*, *and zur*. The down-regulated *lasR* could explain the decreased expression of genes related to H2-T6SS, H3-T6SS, T2SS and pyocyanin, and an increase in the H1-T6SS (Venturi, 2006; Lesic et al., 2009). Other virulence factors affected by H3-T6SS can either directly or indirectly attributed to these regulators. ClpV is a cytoplasmic AAA+ ATPase protein and it is recently found to be localized at discrete and dynamic foci in the cell (Basler and Mekalanos, 2012; Lennings et al., 2019), suggesting additional roles beyond providing energy for T6SSs for this protein. The energy intensive function of T6SS depends on such an ATPase. It is tempting to speculate that, once *clpV3* is deleted, the balance of ATP pool of the cell might be disrupted. ATP dependent synthesis of signaling molecules such as cAMP, as well as the quorum sensing signal molecules might have been affected as a result, which in turn would affect the downstream of the regulatory pathways, contributing to the altered pathogenicity associated phenotypes including biofilm formation, proteolytic activity, pyocyanin production, swarming and swimming motilities. T3SS is an ATP-dependent protein secretion system. T3SS activity is energy intensive and potentially influenced by cellular energy level; therefore, any changes in the cellular ATP pool could theoretically affect this system.

As multiple virulence factors changed significantly in the H3-T6SS mutant, we examined the relevance of such changes *in vivo* using a well-established *G. mellonella* infection model. *G. mellonella* larvae were infected by both the wild-type PAO1 and PAO1(Δ*clpV3*) and the survival rates were compared. The significantly decreased mortality rate in PAO1(Δ*clpV3*), when compared to the PAO1, confirmed that H3-T6SS plays an important role in the *in vivo* pathogenicity of *P. aeruginosa*. The lower expressed virulence including T3SS, pyocyanin production, and elastase may account for the decreased mortality. Because it is possible that H3-T6SS may be directly involved in killing the host, more studies are required to differentiate the direct contributions of the impairment of H3-T6SS in the decreased pathogenicity against *G. mellonella*.

Collectively, the results obtained in this study suggest that H3-T6SS in *P. aeruginosa* plays far more diverse roles and affects many more genes rather than just those related to T6SS, revealing a surprisingly complex connection of this system with other secretions systems and pathogenicity. These results add to the limited pool of knowledge in this field and suggest that T6SS might be a potential therapeutic target against *P. aeruginosa* infections.

## Data Availability Statement

RNA-seq data can be found in the NCBI database, the data was assigned a accession number of GSE148116.

## Author Contributions

KD directed and designed the research. YL, PZ, and LC conducted the experiments. YL and AB analyzed the data. YL and KD wrote the manuscript.

## Conflict of Interest

The authors declare that the research was conducted in the absence of any commercial or financial relationships that could be construed as a potential conflict of interest.

## Footnotes

1 https://github.com/OpenGene/fastp

2 http://www.r-project.org/
