# Supplementary material for: ClpV3 of the H3-Type VI Secretion System (H3-T6SS) Affects Multiple Virulence Factors in Pseudomonas aeruginosa
Source: Front Microbiol. 2020 May 29;11:1096. doi: 10.3389/fmicb.2020.01096 (PMC7273116; doi:10.3389/fmicb.2020.01096)
Supplement: Supplementary file 1 [file Data_Sheet_1.docx]

Supplementary Material

**
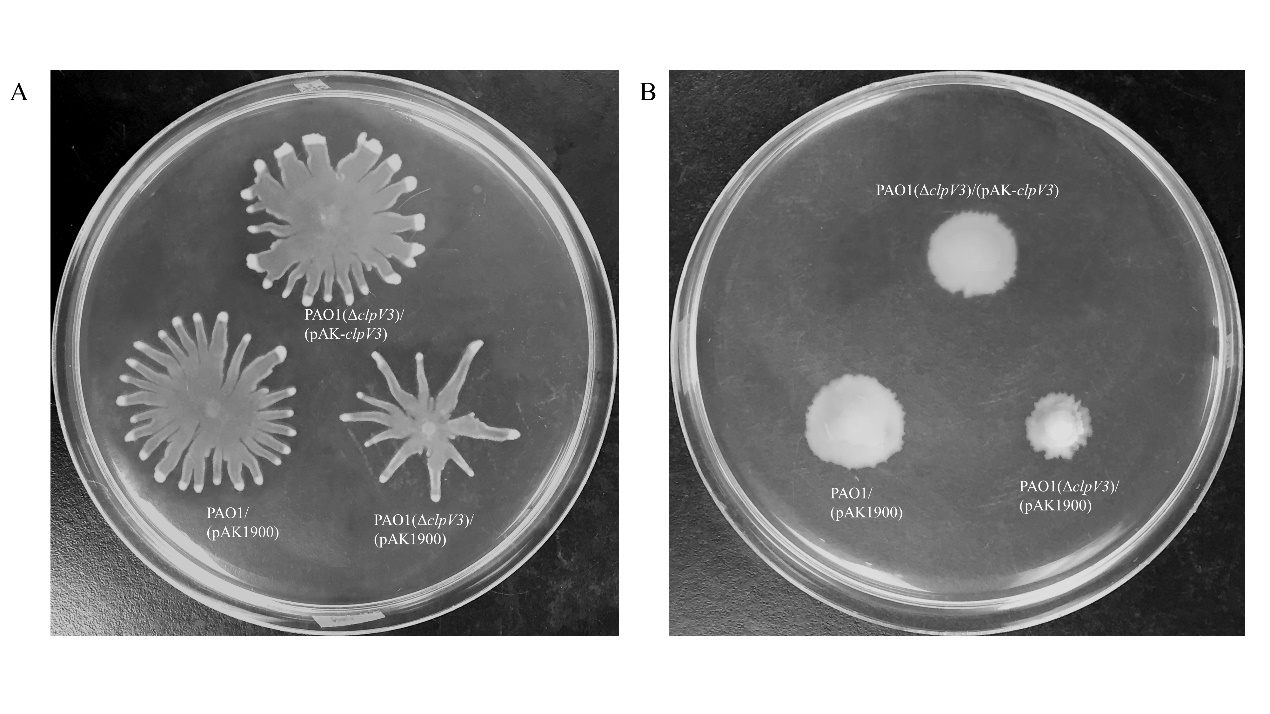
**

**FIGURE S1:** Effect of *clpV3* mutation on swarming (A) and swimming (B) motilities in *P. aeruginosa*.

**TABLE S1:** RNA-Sequencing analysis of differentially expressed genes in PAO1 vs PAO1(Δ *clpV3*).

| **Gene ID** | **Gene name** | **Gene Product** | **log2(fold change)** | ***q*-value** |
| --- | --- | --- | --- | --- |
| **Down-regulated genes** | | | | |
| PA0044 | *exoT* | exoenzyme T | -2.5530 | 0.0000 |
| PA0116 | PA0116 | hypothetical protein | -1.0535 | 0.0378 |
| PA0122 | *rahU* | rahU | -1.8475 | 0.0000 |
| PA0156 | *triA* | Resistance-Nodulation-Cell Division (RND) triclosan efflux membrane fusion protein, TriA | -1.3615 | 0.0027 |
| PA0180 | *cttP* | chemotactic transducer for trichloroethylene [positive chemotaxis], CttP | -1.2256 | 0.0085 |
| PA0195 | *pntAA* | putative NAD(P) transhydrogenase, subunit alpha part 1 | -1.0713 | 0.0100 |
| PA0196 | *pntB* | pyridine nucleotide transhydrogenase, beta subunit | -1.4923 | 0.0115 |
| PA0208 | *mdcA* | malonate decarboxylase alpha subunit | -1.8294 | 0.0007 |
| PA0211 | *mdcD* | malonate decarboxylase beta subunit | -2.7543 | 0.0012 |
| PA0315 | PA0315 | hypothetical protein | -1.1591 | 0.0055 |
| PA0355 | *pfpI* | protease PfpI | -1.3699 | 0.0228 |
| PA0382 | *micA* | DNA mismatch repair protein MicA | -14.4519 | 0.0031 |
| PA0393 | *proC* | pyrroline-5-carboxylate reductase | -1.4602 | 0.0372 |
| PA0446 | PA0446 | conserved hypothetical protein | -3.6646 | 0.0000 |
| PA0447 | *gcdH* | glutaryl-CoA dehydrogenase | -3.6065 | 0.0000 |
| PA0506 | PA0506 | probable acyl-CoA dehydrogenase | -1.0633 | 0.0041 |
| PA0534 | *pauB1* | FAD-dependent oxidoreductase | -2.4606 | 0.0000 |
| PA0547 | PA0547 | probable transcriptional regulator | -1.1833 | 0.0058 |
| PA0554 | PA0554 | hypothetical protein | -1.1992 | 0.0058 |
| PA0565 | PA0565 | conserved hypothetical protein | -1.0504 | 0.0466 |
| PA0779 | *asrA* | AsrA | -1.3426 | 0.0218 |
| PA0792 | *prpD* | propionate catabolic protein PrpD | -1.1654 | 0.0085 |
| PA0807 | *ampDh3* | AmpDh3 | -1.8057 | 0.0000 |
| PA0843 | *plcR* | phospholipase accessory protein PlcR precursor | -13.9806 | 0.0223 |
| PA0852 | *cbpD* | chitin-binding protein CbpD precursor | -2.8163 | 0.0000 |
| PA0852.1 | PA0852.1 | Uncharacterized protein | -2.8734 | 0.0000 |
| PA0905 | *rsmA* | RsmA | -1.7826 | 0.0000 |
| PA1118 | PA1118 | hypothetical protein | -1.1718 | 0.0428 |
| PA1213 | PA1213 | hypothetical protein | -2.7160 | 0.0203 |
| PA1214 | PA1214 | hypothetical protein | -3.2877 | 0.0008 |
| PA1215 | PA1215 | hypothetical protein | -2.4742 | 0.0003 |
| PA1216 | PA1216 | hypothetical protein | -2.7512 | 0.0000 |
| PA1217 | PA1217 | probable 2-isopropylmalate synthase | -2.8765 | 0.0000 |
| PA1244 | *qslA* | QslA | -1.1495 | 0.0011 |
| PA1245 | *aprX* | AprX | -1.2154 | 0.0225 |
| PA1249 | *aprA* | alkaline metalloproteinase precursor | -2.1341 | 0.0000 |
| PA1323 | PA1323 | hypothetical protein | -1.4144 | 0.0027 |
| PA1324 | PA1324 | hypothetical protein | -1.3400 | 0.0001 |
| PA1392 | PA1392 | hypothetical protein | -1.1447 | 0.0380 |
| PA1592 | PA1592 | hypothetical protein | -1.2590 | 0.0008 |
| PA1597 | PA1597 | hypothetical protein | -2.3095 | 0.0000 |
| PA1605 | PA1605 | hypothetical protein | -2.1347 | 0.0176 |
| PA1706 | *pcrV* | type III secretion protein PcrV | -3.1781 | 0.0000 |
| PA1708 | *popB* | translocator protein PopB | -2.8531 | 0.0000 |
| PA1709 | *popD* | Translocator outer membrane protein PopD precursor | -3.3764 | 0.0000 |
| PA1710 | *exsC* | ExsC, exoenzyme S synthesis protein C precursor. | -2.9249 | 0.0000 |
| PA1712 | *exsB* | exoenzyme S synthesis protein B | -2.6450 | 0.0001 |
| PA1763 | PA1763 | hypothetical protein | -2.5713 | 0.0385 |
| PA1784 | PA1784 | hypothetical protein | -1.5900 | 0.0000 |
| PA1835 | PA1835 | hypothetical protein | -1.3774 | 0.0159 |
| PA1852 | PA1852 | hypothetical protein | -1.5681 | 0.0000 |
| PA1870 | PA1870 | hypothetical protein | -1.5424 | 0.0428 |
| PA1874 | PA1874 | hypothetical protein | -2.0537 | 0.0000 |
| PA1875 | *opmL* | probable outer membrane protein precursor | -1.4506 | 0.0002 |
| PA1876 | PA1876 | probable ATP-binding/permease fusion ABC transporter | -1.7071 | 0.0000 |
| PA1877 | PA1877 | probable secretion protein | -1.7741 | 0.0002 |
| PA1888 | PA1888 | hypothetical protein | -1.2618 | 0.0002 |
| PA1894 | PA1894 | hypothetical protein | -1.3454 | 0.0267 |
| PA1899 | *phzA2* | probable phenazine biosynthesis protein | -2.4911 | 0.0000 |
| PA1900 | *phzB2* | probable phenazine biosynthesis protein | -2.4911 | 0.0000 |
| PA1901 | *phzC2* | phenazine biosynthesis protein PhzC | -1.8864 | 0.0008 |
| PA1904 | *phzF2* | probable phenazine biosynthesis protein | -1.4135 | 0.0012 |
| PA1914 | *hvn* | conserved hypothetical protein | -2.9020 | 0.0000 |
| PA1930 | PA1930 | probable chemotaxis transducer | -1.2493 | 0.0004 |
| PA2003 | *bdhA* | 3-hydroxybutyrate dehydrogenase | -1.0463 | 0.0246 |
| PA2069 | PA2069 | probable carbamoyl transferase | -2.6546 | 0.0001 |
| PA2144 | *glgP* | glycogen phosphorylase | -2.2634 | 0.0006 |
| PA2147 | *katE* | catalase HPII | -3.4277 | 0.0000 |
| PA2160 | *glgX* | probable glycosyl hydrolase | -2.2722 | 0.0000 |
| PA2163 | PA2163 | hypothetical protein | -12.2703 | 0.0207 |
| PA2165 | *glgA* | probable glycogen synthase | -3.7268 | 0.0000 |
| PA2166 | PA2166 | hypothetical protein | -2.0644 | 0.0009 |
| PA2167 | PA2167 | hypothetical protein | -2.5149 | 0.0251 |
| PA2171 | PA2171 | hypothetical protein | -2.1917 | 0.0000 |
| PA2172 | PA2172 | hypothetical protein | -4.3054 | 0.0001 |
| PA2190 | PA2190 | conserved hypothetical protein | -1.8855 | 0.0453 |
| PA2300 | *chiC* | chitinase | -2.5202 | 0.0000 |
| PA2365 | *hsiB3* | HsiB3 | -1.5761 | 0.0001 |
| PA2366 | *hsiC3* | HsiC3 | -1.3580 | 0.0004 |
| PA2372 | PA2372 | hypothetical protein | -1.1837 | 0.0010 |
| PA2373 | *vgrG3* | VgrG3 | -1.2686 | 0.0011 |
| PA2414 | PA2414 | L-sorbosone dehydrogenase | -1.7648 | 0.0000 |
| PA2423 | PA2423 | hypothetical protein | -1.4646 | 0.0004 |
| PA2448 | PA2448 | hypothetical protein | -1.2609 | 0.0401 |
| PA2566 | PA2566 | conserved hypothetical protein | -1.6619 | 0.0000 |
| PA2570 | *lecA* | LecA | -1.9816 | 0.0000 |
| PA2571 | PA2571 | probable two-component sensor | -1.0829 | 0.0063 |
| PA2622 | *cspD* | cold-shock protein CspD | -1.1668 | 0.0022 |
| PA2746 | PA2746 | hypothetical protein | -1.3284 | 0.0010 |
| PA2751 | *yfkH* | conserved hypothetical protein | -2.0256 | 0.0091 |
| PA2896 | *sbrI* | SbrI | -2.0834 | 0.0352 |
| PA2939 | *pepB* | probable aminopeptidase | -2.0750 | 0.0000 |
| PA2953 | PA2953 | electron transfer flavoprotein-ubiquinone oxidoreductase | -1.8093 | 0.0000 |
| PA3032 | *snr1* | cytochrome c Snr1 | -2.0116 | 0.0003 |
| PA3190 | *gltB* | probable binding protein component of ABC sugar transporter | -1.3850 | 0.0003 |
| PA3250 | PA3250 | hypothetical protein | -1.1506 | 0.0055 |
| PA3261 | PA3261 | hypothetical protein | -1.9308 | 0.0021 |
| PA3274 | PA3274 | hypothetical protein | -1.9108 | 0.0488 |
| PA3286 | PA3286 | beta-acetoacetyl-acyl carrier protein synthase | -1.3881 | 0.0021 |
| PA3346 | *hsbR* | HptB-dependent secretion and biofilm regulator HsbR | -1.2943 | 0.0009 |
| PA3361 | *lecB* | fucose-binding lectin PA-IIL | -1.0107 | 0.0237 |
| PA3459 | *asnB* | probable glutamine amidotransferase | -1.1751 | 0.0146 |
| PA3460 | PA3460 | probable acetyltransferase | -1.5690 | 0.0007 |
| PA3461 | *yhfE* | conserved hypothetical protein | -1.2133 | 0.0371 |
| PA3478 | *rhlB* | rhamnosyltransferase chain B | -1.6768 | 0.0002 |
| PA3479 | *rhlA* | rhamnosyltransferase chain A | -2.1729 | 0.0000 |
| PA3520 | PA3520 | hypothetical protein | -2.7924 | 0.0000 |
| PA3676 | *mexK* | MexK | -1.4707 | 0.0008 |
| PA3677 | *mexJ* | MexJ | -1.5277 | 0.0281 |
| PA3692 | *lptF* | Lipotoxon F, LptF | -1.3403 | 0.0022 |
| PA3724 | *lasB* | elastase LasB | -1.4427 | 0.0000 |
| PA3796 | PA3796 | hypothetical protein | -1.1639 | 0.0324 |
| PA3841 | *exoS* | exoenzyme S | -2.4906 | 0.0000 |
| PA3842 | *spcS* | specific Pseudomonas chaperone for ExoS, SpcS | -2.1512 | 0.0336 |
| PA3952 | PA3952 | hypothetical protein | -1.9416 | 0.0192 |
| PA3986 | PA3986 | hypothetical protein | -1.3436 | 0.0004 |
| PA4078 | PA4078 | probable nonribosomal peptide synthetase | -1.8732 | 0.0000 |
| PA4175 | *piv* | protease IV | -2.7490 | 0.0000 |
| PA4210 | *phzA1* | probable phenazine biosynthesis protein | -1.4135 | 0.0000 |
| PA4215 | *phzF1* | probable phenazine biosynthesis protein | -1.4135 | 0.0012 |
| PA4216 | *phzG1* | probable pyridoxamine 5'-phosphate oxidase | -1.4922 | 0.0009 |
| PA4294 | PA4294 | hypothetical protein | -1.0917 | 0.0101 |
| PA4297 | *tadG* | TadG | -1.2230 | 0.0089 |
| PA4299 | *tadD* | TadD | -1.3127 | 0.0043 |
| PA4301 | *tadB* | TadB | -1.4851 | 0.0116 |
| PA4302 | *tadA* | TadA ATPase | -1.6774 | 0.0000 |
| PA4303 | *tadZ* | TadZ | -1.9504 | 0.0000 |
| PA4304 | *rcpA* | RcpA | -1.8171 | 0.0000 |
| PA4305 | *rcpC* | RcpC | -1.9208 | 0.0000 |
| PA4306 | *flp* | Type IVb pilin, Flp | -1.5632 | 0.0000 |
| PA4377 | PA4377 | hypothetical protein | -2.5578 | 0.0000 |
| PA4385 | *groEL* | GroEL protein | -1.0614 | 0.0069 |
| PA4386 | *groES* | GroES protein | -1.2881 | 0.0045 |
| PA4395 | *yajQ* | conserved hypothetical protein | -1.0575 | 0.0111 |
| PA4462 | *rpoN* | RNA polymerase sigma-54 factor | -1.0902 | 0.0128 |
| PA4542 | *clpB* | ClpB protein | -1.1494 | 0.0008 |
| PA4590 | *pra* | protein activator | -2.3513 | 0.0010 |
| PA4648 | *cupE1* | Pilin subunit CupE1 | -2.2498 | 0.0000 |
| PA4649 | *cupE2* | Pilin subunit CupE2 | -1.1284 | 0.0325 |
| PA4679 | PA4679 | hypothetical protein | -2.1489 | 0.0426 |
| PA4738 | *yjbJ* | conserved hypothetical protein | -1.3133 | 0.0130 |
| PA4759 | *dapB* | dihydrodipicolinate reductase | -1.3311 | 0.0036 |
| PA4760 | *dnaJ* | DnaJ protein | -1.2571 | 0.0009 |
| PA4761 | *dnaK* | DnaK protein | -1.6744 | 0.0001 |
| PA4762 | *grpE* | heat shock protein GrpE | -1.7749 | 0.0003 |
| PA4848 | *accC* | biotin carboxylase | -1.0139 | 0.0205 |
| PA4876 | *osmE* | osmotically inducible lipoprotein OsmE | -1.3389 | 0.0002 |
| PA4877 | PA4877 | hypothetical protein | -1.4711 | 0.0281 |
| PA4880 | PA4880 | probable bacterioferritin | -1.1064 | 0.0038 |
| PA5053 | *hslV* | heat shock protein HslV | -1.6420 | 0.0070 |
| PA5058 | *phaC2* | poly(3-hydroxyalkanoic acid) synthase 2 | -1.1030 | 0.0111 |
| PA5161 | *rmlB* | dTDP-D-glucose 4,6-dehydratase | -1.0824 | 0.0171 |
| PA5285 | *sutA* | SutA | -1.0403 | 0.0043 |
| PA5312 | *pauC* | Aldehyde dehydrogenase | -1.1018 | 0.0267 |
| PA5313 | *gabT2* | Transaminase | -2.5778 | 0.0002 |
| PA5348 | PA5348 | probable DNA-binding protein | -1.8635 | 0.0000 |
| PA5421 | *fdhA* | glutathione-independent formaldehyde dehydrogenase | -1.4511 | 0.0068 |
| PA5527 | PA5527 | hypothetical protein | -1.2056 | 0.0007 |
| PA5546 | *Rv3720* | conserved hypothetical protein | -1.1984 | 0.0028 |
| **Up-regulated genes** | | | | |
| PA0008 | *glyS* | glycyl-tRNA synthetase beta chain | 1.2757 | 0.0402 |
| PA0070 | *tagQ1* | TagQ1 | 1.5573 | 0.0000 |
| PA0074 | *ppkA* | serine/threonine protein kinase PpkA | 1.4318 | 0.0040 |
| PA0084 | *tssC1* | TssC1 | 1.2002 | 0.0028 |
| PA0085 | *hcp1* | Hcp1 | 1.2362 | 0.0049 |
| PA0141 | PA0141 | conserved hypothetical protein | 1.1107 | 0.0018 |
| PA0162 | *opdC* | histidine porin OpdC | 2.9283 | 0.0024 |
| PA0291 | *oprE* | Anaerobically-induced outer membrane porin OprE precursor | 1.4581 | 0.0001 |
| PA0305 | *hacB* | acylhomoserine lactone acylase B | 1.6911 | 0.0163 |
| PA0412 | *pilK* | methyltransferase PilK | 1.4112 | 0.0150 |
| PA0423 | *pasP* | PasP | 1.0804 | 0.0048 |
| PA0509 | *nirN* | NirN | 1.2464 | 0.0130 |
| PA0510 | *nirE* | NirE | 1.7612 | 0.0002 |
| PA0511 | *nirJ* | heme d1 biosynthesis protein NirJ | 1.3358 | 0.0138 |
| PA0512 | *nirH* | NirH | 2.6891 | 0.0192 |
| PA0514 | *nirL* | heme d1 biosynthesis protein NirL | 1.9597 | 0.0004 |
| PA0515 | *nirD* | probable transcriptional regulator | 2.6497 | 0.0000 |
| PA0524 | *norB* | nitric-oxide reductase subunit B | 1.5158 | 0.0132 |
| PA0545 | PA0545 | hypothetical protein | 1.3588 | 0.0002 |
| PA0594 | *surA* | peptidyl-prolyl cis-trans isomerase SurA | 1.1249 | 0.0146 |
| PA0608 | *gph* | probable phosphoglycolate phosphatase | 2.2296 | 0.0044 |
| PA0609 | *trpE* | anthranilate synthetase component I | 5.8122 | 0.0000 |
| PA0649 | *trpG* | anthranilate synthase component II | 4.6206 | 0.0000 |
| PA0650 | *trpD* | anthranilate phosphoribosyltransferase | 2.3761 | 0.0000 |
| PA0651 | *trpC* | indole-3-glycerol-phosphate synthase | 3.7272 | 0.0000 |
| PA0654 | *speD* | S-adenosylmethionine decarboxylase proenzyme | 1.5215 | 0.0426 |
| PA0713 | PA0713 | hypothetical protein | 1.1912 | 0.0122 |
| PA0723 | *coaB* | coat protein B of bacteriophage Pf1 | 2.4807 | 0.0426 |
| PA0836 | *ackA* | acetate kinase | 1.1327 | 0.0103 |
| PA0840 | PA0840 | probable oxidoreductase | 3.4307 | 0.0256 |
| PA0867 | *mliC* | membrane-bound lysozyme inhibitor of c-type lysozyme MliC | 1.3537 | 0.0014 |
| PA0887 | *acsA* | acetyl-coenzyme A synthetase | 1.5696 | 0.0000 |
| PA0936 | *lpxO2* | lipopolysaccharide biosynthetic protein LpxO2 | 1.4069 | 0.0141 |
| PA1001 | *phnA* | anthranilate synthase component I | 1.1296 | 0.0064 |
| PA1045 | PA1045 | hypothetical protein | 2.0480 | 0.0183 |
| PA1050 | *yeaG* | conserved hypothetical protein | 1.2123 | 0.0205 |
| PA1091 | *fgtA* | flagellar glycosyl transferase, FgtA | 1.1002 | 0.0043 |
| PA1155 | *nrdB* | NrdB, tyrosyl radical-harboring component of class Ia ribonucleotide reductase | 1.3785 | 0.0001 |
| PA1183 | *dctA* | C4-dicarboxylate transport protein | 1.4512 | 0.0247 |
| PA1187 | *lcaD* | probable acyl-CoA dehydrogenase | 8.6434 | 0.0000 |
| PA1338 | *ggt* | gamma-glutamyltranspeptidase precursor | 1.2362 | 0.0032 |
| PA1429 | PA1429 | probable cation-transporting P-type ATPase | 1.0788 | 0.0055 |
| PA1555 | *ccoP2* | Cytochrome c oxidase, cbb3-type, CcoP subunit | 1.6209 | 0.0000 |
| PA1555.1 | *ccoQ2* | Cytochrome c oxidase, cbb3-type, CcoQ subunit | 1.8723 | 0.0002 |
| PA1556 | *ccoO2* | Cytochrome c oxidase, cbb3-type, CcoO subunit | 1.4674 | 0.0002 |
| PA1557 | *ccoN2* | Cytochrome c oxidase, cbb3-type, CcoN subunit | 1.1944 | 0.0085 |
| PA1561 | *aer* | aerotaxis receptor Aer | 1.5175 | 0.0000 |
| PA1586 | *sucB* | dihydrolipoamide succinyltransferase (E2 subunit) | 1.0475 | 0.0117 |
| PA1589 | *sucD* | succinyl-CoA synthetase alpha chain | 1.2040 | 0.0006 |
| PA1673 | PA1673 | hypothetical protein | 1.2481 | 0.0054 |
| PA1746 | PA1746 | hypothetical protein | 1.1540 | 0.0200 |
| PA1766 | PA1766 | hypothetical protein | 1.3225 | 0.0228 |
| PA1767 | PA1767 | hypothetical protein | 2.0799 | 0.0022 |
| PA1795 | *cysS* | cysteinyl-tRNA synthetase | 1.0785 | 0.0336 |
| PA1828 | PA1828 | probable short-chain dehydrogenase | 1.5649 | 0.0137 |
| PA1964 | *ybiT* | probable ATP-binding component of ABC transporter | 1.5280 | 0.0159 |
| PA2014 | *liuB* | methylcrotonyl-CoA carboxylase, beta-subunit | 1.2390 | 0.0112 |
| PA2015 | *liuA* | putative isovaleryl-CoA dehydrogenase | 1.0429 | 0.0129 |
| PA2036 | PA2036 | hypothetical protein | 2.5509 | 0.0242 |
| PA2119 | *adh* | alcohol dehydrogenase (Zn-dependent) | 1.0865 | 0.0056 |
| PA2126.1 | *cgrB* | cupA gene regulator B, CgrB | 1.8688 | 0.0380 |
| PA2127 | *cgrA* | cupA gene regulator A, CgrA | 2.2006 | 0.0000 |
| PA2128 | *cupA1* | fimbrial subunit CupA1 | 1.8060 | 0.0000 |
| PA2195 | *hcnC* | hydrogen cyanide synthase HcnC | 1.2727 | 0.0088 |
| PA2259 | *ptxS* | transcriptional regulator PtxS | 1.1335 | 0.0240 |
| PA2260 | *kguE* | hypothetical protein | 1.5636 | 0.0270 |
| PA2323 | *gapN* | GapN | 2.2767 | 0.0101 |
| PA2453 | PA2453 | hypothetical protein | 3.9945 | 0.0380 |
| PA2462 | PA2462 | hypothetical protein | 1.0219 | 0.0053 |
| PA2463 | PA2463 | hypothetical protein | 3.2422 | 0.0109 |
| PA2501 | PA2501 | hypothetical protein | 2.5236 | 0.0001 |
| PA2508 | *catC* | muconolactone delta-isomerase | 4.7085 | 0.0029 |
| PA2511 | *antR* | AntR | 4.1369 | 0.0000 |
| PA2512 | *antA* | anthranilate dioxygenase large subunit | 17.1692 | 0.0000 |
| PA2513 | *antB* | anthranilate dioxygenase small subunit | 4.4144 | 0.0000 |
| PA2514 | *antC* | anthranilate dioxygenase reductase | 14.6830 | 0.0000 |
| PA2555 | PA2555 | probable AMP-binding enzyme | 1.4394 | 0.0048 |
| PA2630 | *ycfD* | conserved hypothetical protein | 1.1589 | 0.0336 |
| PA2682 | PA2682 | conserved hypothetical protein | 2.6721 | 0.0021 |
| PA2683.1 | *tsi5* | Tsi5 | 2.0542 | 0.0182 |
| PA2684 | *tse5* | Tse5 | 1.5962 | 0.0012 |
| PA2709 | *cysK* | cysteine synthase A | 1.0860 | 0.0374 |
| PA2943 | PA2943 | phospho-2-dehydro-3-deoxyheptonate aldolase | 6.6349 | 0.0000 |
| PA2996 | *nqrD* | Na+-translocating NADH:uniquinone oxidoreductase subunit Nqr4 | 2.1321 | 0.0380 |
| PA2998 | *nqrB* | Na+-translocating NADH:ubiquinone oxidoreductase subunit Nrq2 | 1.7576 | 0.0278 |
| PA3000 | *aroP1* | aromatic amino acid transport protein AroP1 | 2.1643 | 0.0337 |
| PA3054 | PA3054 | hypothetical protein | 1.1393 | 0.0127 |
| PA3081 | PA3081 | conserved hypothetical protein | 2.5927 | 0.0085 |
| PA3186 | *oprB* | Glucose/carbohydrate outer membrane porin OprB precursor | 1.3433 | 0.0380 |
| PA3233 | PA3233 | hypothetical protein | 1.8208 | 0.0239 |
| PA3278 | PA3278 | hypothetical protein | 1.1173 | 0.0136 |
| PA3337 | *rfaD* | ADP-L-glycero-D-mannoheptose 6-epimerase | 1.6134 | 0.0001 |
| PA3364 | *amiC* | aliphatic amidase expression-regulating protein | 1.0861 | 0.0183 |
| PA3391 | *nosR* | regulatory protein NosR | 1.5980 | 0.0096 |
| PA3392 | *nosZ* | nitrous-oxide reductase precursor | 1.4801 | 0.0002 |
| PA3458 | PA3458 | probable transcriptional regulator | 1.3725 | 0.0001 |
| PA3581 | *glpF* | glycerol uptake facilitator protein | 1.9235 | 0.0000 |
| PA3582 | *glpK* | glycerol kinase | 2.4626 | 0.0000 |
| PA3583 | *glpR* | glycerol-3-phosphate regulon repressor | 1.9841 | 0.0132 |
| PA3584 | *glpD* | glycerol-3-phosphate dehydrogenase | 3.0293 | 0.0000 |
| PA3694 | PA3694 | hypothetical protein | 3.3429 | 0.0386 |
| PA3728 | PA3728 | hypothetical protein | 1.1960 | 0.0150 |
| PA3735 | *thrC* | threonine synthase | 2.0682 | 0.0242 |
| PA3753 | *fbp* | conserved hypothetical protein | 1.0731 | 0.0444 |
| PA3763 | *purL* | phosphoribosylformylglycinamidine synthase | 1.0320 | 0.0489 |
| PA3789 | PA3789 | hypothetical protein | 1.2466 | 0.0238 |
| PA3790 | *oprC* | Putative copper transport outer membrane porin OprC precursor | 1.7807 | 0.0000 |
| PA3802 | *hisS* | histidyl-tRNA synthetase | 1.1102 | 0.0091 |
| PA3871 | *nifM* | probable peptidyl-prolyl cis-trans isomerase, PpiC-type | 4.3030 | 0.0088 |
| PA3874 | *narH* | respiratory nitrate reductase beta chain | 1.0761 | 0.0402 |
| PA3875 | *narG* | respiratory nitrate reductase alpha chain | 1.0580 | 0.0200 |
| PA3879 | *narL* | two-component response regulator NarL | 1.2909 | 0.0256 |
| PA3922 | PA3922 | conserved hypothetical protein | 1.3450 | 0.0065 |
| PA3923 | PA3923 | hypothetical protein | 1.1445 | 0.0068 |
| PA4133 | *cytN* | cytochrome c oxidase subunit (cbb3-type) | 1.0541 | 0.0240 |
| PA4138 | *tyrS* | tyrosyl-tRNA synthetase | 6.7201 | 0.0000 |
| PA4259 | *rpsS* | 30S ribosomal protein S19 | 1.0270 | 0.0100 |
| PA4260 | *rplB* | 50S ribosomal protein L2 | 1.0757 | 0.0162 |
| PA4261 | *rplW* | 50S ribosomal protein L23 | 1.6174 | 0.0065 |
| PA4262 | *rplD* | 50S ribosomal protein L4 | 1.1736 | 0.0032 |
| PA4269 | *rpoC* | DNA-directed RNA polymerase beta* chain | 1.1103 | 0.0044 |
| PA4270 | *rpoB* | DNA-directed RNA polymerase beta chain | 1.1144 | 0.0039 |
| PA4389 | PA4389 | 3-oxacyl-acyl carrier protein reductase | 1.5443 | 0.0035 |
| PA4410 | *ddlB* | D-alanine--D-alanine ligase | 1.1661 | 0.0228 |
| PA4415 | *mraY* | phospho-N-acetylmuramoyl-pentapeptide-transferase | 1.1803 | 0.0270 |
| PA4464 | *ptsN* | nitrogen regulatory IIA protein | 1.1806 | 0.0040 |
| PA4489 | *magD* | MagD | 1.3394 | 0.0001 |
| PA4560 | *ileS* | isoleucyl-tRNA synthetase | 1.1187 | 0.0150 |
| PA4587 | *ccpR* | cytochrome c551 peroxidase precursor | 1.9112 | 0.0000 |
| PA4605 | *ybdD* | conserved hypothetical protein | 3.0016 | 0.0004 |
| PA4696 | *ilvI* | acetolactate synthase large subunit | 1.1887 | 0.0141 |
| PA4714 | PA4714 | conserved hypothetical protein | 1.1872 | 0.0385 |
| PA4803 | PA4803 | hypothetical protein | 2.1310 | 0.0153 |
| PA4843 | *gcbA* | GcbA | 1.2239 | 0.0010 |
| PA4919 | *pncB1* | nicotinate phosphoribosyltransferase | 2.0119 | 0.0001 |
| PA5016 | *aceF* | dihydrolipoamide acetyltransferase | 1.2457 | 0.0006 |
| PA5080 | *pip* | prolyl aminopeptidase | 1.2495 | 0.0141 |
| PA5106 | PA5106 | conserved hypothetical protein | 2.0603 | 0.0021 |
| PA5113 | PA5113 | hypothetical protein | 1.5203 | 0.0426 |
| PA5153 | PA5153 | amino acid (lysine/arginine/ornithine/histidine/octopine) ABC transporter periplasmic binding protein | 1.7061 | 0.0002 |
| PA5169 | *dctM* | DctM | 2.0813 | 0.0203 |
| PA5201 | *tex* | conserved hypothetical protein | 1.7052 | 0.0162 |
| PA5224 | *pepP* | aminopeptidase P | 1.1597 | 0.0045 |
| PA5236 | *ubiB* | probable aromatic hydrocarbon reductase | 2.7980 | 0.0197 |
| PA5332 | *crc* | catabolite repression control protein | 1.1841 | 0.0017 |
| PA5339 | PA5339 | conserved hypothetical protein | 1.0509 | 0.0205 |
| PA5353 | *glcF* | glycolate oxidase subunit GlcF | 2.5931 | 0.0013 |
| PA5358 | *ubiA* | 4-hydroxybenzoate-octaprenyl transferase | 13.5537 | 0.0262 |
| PA5446 | PA5446 | hypothetical protein | 1.3963 | 0.0074 |
| PA5456 | PA5456 | putative glycosyltransferase | 1.7859 | 0.0096 |
| PA5460 | PA5460 | hypothetical protein | 4.9083 | 0.0369 |
| PA5549 | *glmS* | glucosamine--fructose-6-phosphate aminotransferase | 1.9200 | 0.0012 |

**TABLE S2:** Differential expression of protein secretion systems, pyocyanin synthesis and transcriptional regulators in PAO1(Δ*clpV3*) shown by RNA-Seq data.

| **Gene ID** | **Gene name** | **Gene Product** | **log2(fold change)** | **p-value** |
| --- | --- | --- | --- | --- |
| **T2SS** | | | | |
| PA1871 | *lasA* | LasA protease precursor | -1.0533 | 0.0208 |
| PA3104 | *xcpP* | Secretion protein XcpP | -0.7742 | 0.0127 |
| PA3724 | *lasB* | elastase LasB | -1.4428 | 0.0000 |
| PA4299 | *tadD* | TadD | -1.3127 | 0.0001 |
| PA4301 | *tadB* | TadB | -1.4851 | 0.0005 |
| PA4303 | *TadZ* | TadZ | -1.9504 | 0.0000 |
| PA4304 | *rcpA* | RcpA | -1.8171 | 0.0000 |
| **T3SS** | | | | |
| PA0044 | *exoT* | exoenzyme T | -2.5530 | 0.0000 |
| PA1703 | *pcrD* | type III secretory apparatus protein PcrD | -1.5032 | 0.0176 |
| PA1706 | *pcrV* | Type III secretion protein PcrV | -3.1782 | 0.0000 |
| PA1707 | *pcrH* | Regulatory protein PcrH | -3.5221 | 0.0323 |
| PA1708 | *popB* | Translocator protein PopB | -2.8531 | 0.0000 |
| PA1709 | *popD* | Translocator outer membrane protein PopD precursor | -3.3764 | 0.0000 |
| PA1710 | *exsC* | ExsC, exoenzyme S synthesis protein C precursor. | -2.9249 | 0.0000 |
| PA1712 | *exsB* | exoenzyme S synthesis protein B | -2.6450 | 0.0000 |
| PA1714 | *exsD* | ExsD | -1.5466 | 0.0108 |
| PA1720 | *pscG* | Type III export protein PscG | -14.1200 | 0.0160 |
| PA1722 | *pscI* | Type III export protein PscI | -3.4574 | 0.0410 |
| PA1723 | *pscJ* | Type III export protein PscJ | -1.5880 | 0.0287 |
| PA3841 | *exoS* | ExoS | -2.4906 | 0.0000 |
| **H1-T6SS** | | | | |
| PA0077 | *icmF1* | IcmF1 | 1.2022 | 0.0095 |
| PA0078 | *tssL1* | tssL1 | 1.4687 | 0.0365 |
| PA0080 | *tssJ1* | tssJ1 | 3.7567 | 0.0058 |
| PA0083 | *tssB1* | TssB1 | 1.0625 | 0.0349 |
| PA0084 | *tssC1* | TssC1 | 1.2002 | 0.0001 |
| PA0085 | *hcp1* | Hcp1 | 1.2362 | 0.0002 |
| PA0090 | *clpV1* | ClpV1 | 0.8518 | 0.0082 |
| PA0093 | *tse6* | Tse6 | 2.3809 | 0.0191 |
| PA2685 | *vgrG4* | VgrG4 | 1.3270 | 0.0040 |
| **H2-T6SS** | | | | |
| PA1511 | *vgrG2a* | VgrG2a | -0.7196 | 0.0471 |
| PA1667 | *hsiJ2* | HsiJ2 | -0.8942 | 0.0240 |
| **H3-T6SS** | | | | |
| PA2365 | *hsiB3* | HsiB3 | -1.5761 | 0.0000 |
| PA2366 | *hsiC3* | HsiC3 | -1.3580 | 0.0000 |
| PA2367 | *hcp3* | Hcp3 | -0.9314 | 0.0017 |
| PA2373 | *vgrG3* | VgrG3 | -1.2686 | 0.0000 |
| **Pyocyanin production** | | | | |
| PA1899 | *phzA2* | probable phenazine biosynthesis protein | -2.4911 | 0.0000 |
| PA1900 | *phzB2* | Probable phenazine biosynthesis protein | -2.4911 | 0.0000 |
| PA1901 | *phzC2* | Phenazine biosynthesis protein PhzC | -1.8864 | 0.0000 |
| PA1904 | *phzF2* | Probable phenazine biosynthesis protein | -1.4135 | 0.0000 |
| PA4209 | *phzM* | Probable phenazine-specific methyltransferase | -0.8468 | 0.0306 |
| PA4210 | *phzA1* | probable phenazine biosynthesis protein | -1.4135 | 0.0000 |
| PA4215 | *phzF1* | Probable phenazine biosynthesis protein | -1.4135 | 0.0000 |
| PA4216 | *phzG1* | Probable pyridoxamine 5'-phosphate oxidase | -1.4922 | 0.0000 |
| **Transcriptional regulators** | | | | |
| PA0905 | *rsmA* | RsmA | -1.7826 | 0.0000 |
| PA1430 | *lasR* | Transcriptional regulator LasR | -0.6797 | 0.0140 |
| PA2591 | *vqsR* | VqsR | -0.5895 | 0.0390 |
| PA3678 | *mexL* | MexL | -0.9817 | 0.0214 |
| PA4315 | *mvaT* | Transcriptional regulator MvaT, P16 subunit | -0.9759 | 0.0003 |
| PA5301 | *pauR* | PauR | -1.0308 | 0.0075 |
| PA0964 | *pmpR* | pqsR-mediated PQS regulator, PmpR | 0.9259 | 0.0061 |
| PA2126 | *cgrC* | cupA gene regulator C, CgrC | 1.2416 | 0.0122 |
| PA2126.1 | *cgrB* | cupA gene regulator B, CgrB | 1.8688 | 0.0023 |
| PA2259 | *ptxS* | Transcriptional regulator PtxS | 1.1335 | 0.0012 |
| PA2511 | *antR* | AntR | 4.1369 | 0.0000 |
| PA3879 | *narL* | Two-component response regulator NarL | 1.2909 | 0.0014 |
| PA5499 | *zur* | Zinc uptake regulator, Zur | 1.2630 | 0.0309 |
